# Does the canopy mixing layer model apply to highly flexible aquatic vegetation? Insights from numerical modelling

**DOI:** 10.1007/s10652-016-9482-z

**Published:** 2016-11-02

**Authors:** Timothy I. Marjoribanks, Richard J. Hardy, Stuart N. Lane, Daniel R. Parsons

**Affiliations:** 1 School of Civil and Building Engineering, Loughborough University, Loughborough, LE11 3TU UK; 2 Department of Geography, Durham University, Durham, DH1 3LE UK; 3 Institute of Earth Surface Dynamics, Faculté des géosciences et de l’environnement, Université de Lausanne, 1015 Lausanne, Switzerland; 4 Department of Geography, Environment and Earth Sciences, University of Hull, Hull, HU6 7RX UK

**Keywords:** Eco-hydraulics, Computational fluid dynamics, Vegetation, Biomechanics, Canopy flows, Turbulence

## Abstract

Vegetation is a characteristic feature of shallow aquatic flows such as rivers, lakes and coastal waters. Flow through and above aquatic vegetation canopies is commonly described using a canopy mixing layer analogy which provides a canonical framework for assessing key hydraulic characteristics such as velocity profiles, large-scale coherent turbulent structures and mixing and transport processes for solutes and sediments. This theory is well developed for the case of semi-rigid terrestrial vegetation and has more recently been applied to the case of aquatic vegetation. However, aquatic vegetation often displays key differences in morphology and biomechanics to terrestrial vegetation due to the different environment it inhabits. Here we investigate the effect of plant morphology and biomechanical properties on flow–vegetation interactions through the application of a coupled LES-biomechanical model. We present results from two simulations of aquatic vegetated flows: one assuming a semi-rigid canopy and the other a highly flexible canopy and provide a comparison of the associated flow regimes. Our results show that while both cases display canopy mixing layers, there are also clear differences in the shear layer characteristics and turbulent processes between the two, suggesting that the semi-rigid approximation may not provide a complete representation of flow–vegetation interactions.

## Introduction

Vegetation is a common feature within lowland river environments and influences the functioning of the river system [1]. It acts as an additional source of channel resistance and has been shown to alter bulk flow velocities and conveyance [2–4], generate turbulence through coherent flow structures [5–8], modify sediment transport processes [9–11] and increase habitat diversity [12, 13]. Therefore, a good process understanding of boundary layer flow through and around vegetation is central in predicting the functioning of the fluvial system.

As a result, much research has been conducted into vegetated channels [14]. Our current theoretical understanding of aquatic vegetated flows has been based on our understanding of terrestrial flows through crop fields or forest environments (as reviewed by Finnigan et al. [15]). Terrestrial canopy research led to the development of a canonical theory for canopy mixing layers, based upon classical free shear layers, or mixing layers, which has been used to describe flow through and above terrestrial vegetation canopies [16, 17] (see Sect. 2).

As research into aquatic vegetation canopies has subsequently developed, this theory has been transferred and applied to aquatic environments with much of the terminology associated with terrestrial canopy flows being adopted and adapted for aquatic canopy flows [7, 18]. However, aquatic canopies inhabit very different physical environments to terrestrial canopies. This will alter the force balance between the flow and vegetation and may substantially modify the dynamics of flow–vegetation interactions. As a result, aquatic canopies display differences in morphology and biomechanical properties. Most notably, submerged aquatic macrophytes are often highly flexible and buoyant, which will affect posture and plant-flow interaction [19]. Thus, in this paper we test the hypothesis that there are fundamental differences between aquatic and terrestrial canopy flow structures.

We begin by reviewing general canopy layer theory, which applies to terrestrial vegetation and semi-rigid aquatic canopies, before highlighting the potential differences in highly flexible aquatic canopies. We then use an LES-biomechanical model framework [20] to simulate flow through both an idealised semi-rigid terrestrial-style canopy and a highly flexible canopy more typical of those found within rivers. We apply this model in order to capture the high resolution flow dynamics across the length and breadth of the canopy. Using these data, we characterise both flows within a canopy mixing layer framework and compare the predicted and observed canopy flow variables.

## Canopy mixing layer model for semi-rigid canopies

### Velocity profile

**Fig. 1:**
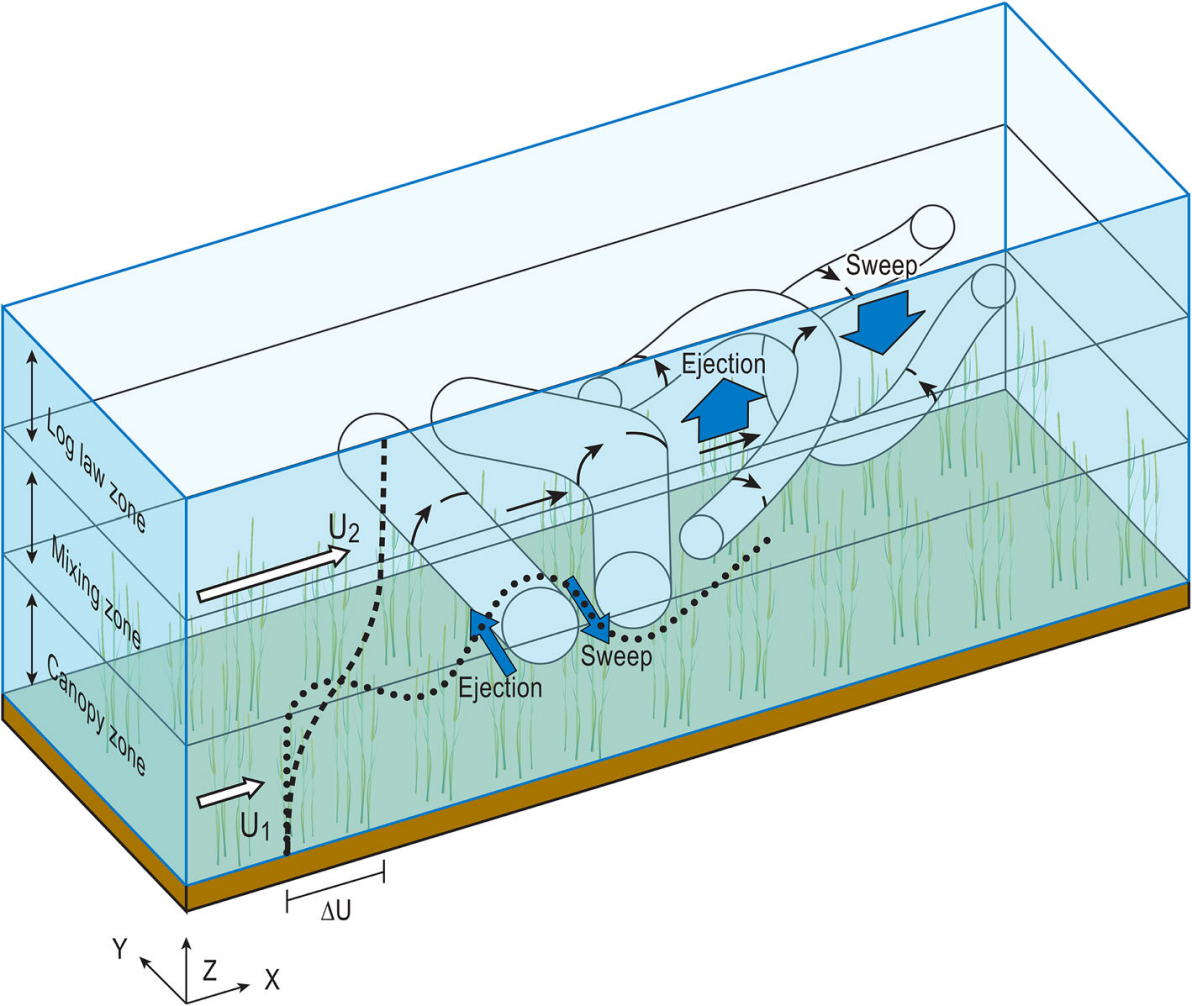
Schematic model of canopy flow. The difference between the velocity within (*U*_1_) and above (*U*_2_) the canopy leads to the development of an inflected velocity profile (*dashed line*). This velocity profile can be split into three zones: (i) the canopy zone, (ii) the mixing zone and (iii) the log law zone. At the inflection point, Kelvin–Helmholtz instabilities form (*dotted line*) which develop into roller vortices which are convected downstream along the canopy top. These vortices are stretched and form pairs of head up (H-U) and head down (H-D) hairpin vortices which induce ejection and sweep events respectively (*blue arrows*). Sweep and ejection events have also been linked to the passage of the roller vortices (*blue arrows*)

Plant canopies act as a porous blockage [21, 22], restricting flow but not preventing it. This porous effect creates two very different velocity regimes: one above and one within the vegetation canopy (*U*_1_ and *U*_2_ in Fig. 1). This leads to the formation of a 3-zone velocity profile [23]. The canopy zone is characterised by a region of low longitudinal velocity and also very low longitudinal velocity gradient in the vertical direction [6, 24]. The log-law zone above the canopy is unaffected by the additional vegetative drag and therefore the velocity follows the typical logarithmic boundary layer profile [25]. Where these two regions meet, there is an inflection point within the velocity profile and a mixing zone forms, with a hyperbolic tangent curve, or S-shaped velocity profile [16, 26, 27]. This velocity profile has been observed both in terrestrial [16] and aquatic canopy flows [5, 7].

### Turbulence structure and characteristics

The turbulence structure of canopy flows can be split into three distinctive length scales, which correspond to the different velocity profile zones, defined as fine-scale wakes, the active mixing layer and the inactive boundary layer [16]. Fine-scale wake turbulence as a result of stem vortex shedding is a key process within the canopy system, controlling the magnitude of the drag discontinuity between the canopy and the flow above, and in turn affecting the scale of canopy mixing layer turbulence [14]. However, despite its importance as a process in defining canopy scale dynamics, stem-scale wake turbulence accounts for only approximately 10 % of the in-canopy turbulence intensity [28]. As it is small-scale in space and time, assuming no backscatter of energy, it will quickly dissipate away into heat [29]. Most canopy flows exist within a larger boundary layer, producing large-scale turbulent structures that scale with the depth of the entire boundary layer. This turbulence will interact with the shear-scale eddies but within the canopy it is less likely to impact on the turbulence statistics and is therefore termed ‘inactive turbulence’ [16].

Instead the active mixing layer turbulence dominates the TKE budget within the canopy [16]. These vortices are generated by the Kelvin–Helmholtz (K–H) instability mechanism as a result of the inflected velocity profile of the free shear layer [30, 31]. The initial inflection point instability evolves and develops into a series of waves which grow downstream before rolling up into distinct, inclined spanwise roller vortices (Fig. 1) [5, 15, 32]. These vortices expand with distance and time until shear production equals canopy dissipation and the vortex reaches its equilibrium size [7, 32, 33].

In between these spanwise rollers, braid regions develop exhibiting high strain rates. Pairs of counter-rotating streamwise rib vortices form in these regions [26] and interact with the roller vortices. Ambient turbulence within the flow then causes pairing of the roller vortices and the interaction between the pair’s vorticity fields causes them to converge and rotate around one another [5, 17]. This eventually leads to the development of pairs of head-up (H-U) and head-down (H-D) vortices which induce sweep and ejection events.

This is a key theory as it links two prominent aspects of turbulence research within canopy flows: the development of K–H instabilities and the occurrence of coherent sweep and ejection motions within the canopy. Following Lu and Willmart [34], sweeps (Q4 events) are defined as events with larger than average downstream velocity and smaller than average vertical (upward) velocity, and ejections (Q2 events) as events with a smaller than average downstream velocity and a larger than average vertical velocity. It is well documented that within canopy flows, sweeps dominate the canopy region and ejections dominate the flow above [24, 32, 35–37]. It is also recognised that these intermittent, high momentum events are responsible for the majority of energy and momentum transfer between the canopy and the flow above [24, 38].

A number of studies of semi-rigid canopies in both terrestrial and aquatic environments have shown the correlation between sweep and ejection events and the passage of canopy roller vortices [8, 17, 23, 24, 39, 40]. In contrast to the theory of Finnigan et al. [17], who relate sweep and ejection events to hairpin vortex formation, other studies hypothesise that sweep and ejection events simply represent manifestations of vortex passage within the velocity signal [39]. Nevertheless, it is clear that mixing layer vortices and sweep and ejection events are two key observable properties of canopy shear layers and that the two are mechanistically linked.

### Plant response and interaction with the flow

Plant motion in response to the flow can be categorised as one of four regimes. These are erect, gently swaying, honami/monami (coherently waving) and prone [6, 18, 41, 42]. The regime of motion observed for a particular canopy will be determined by the biomechanical properties of the vegetation as well as the drag force [32, 43]. While these regimes apply to all canopies, aquatic plants tend to have greater flexibility leading to a greater range of plant motion [6]. The most complex regimes are gently swaying and coherently swaying as these represent dynamic interaction between the flow and canopy. Canopy motion can help absorb momentum from the flow, regulating canopy turbulence [8] and there is also evidence that the natural frequency of the stems can modulate the velocity field and vortex shedding rate [5, 24, 44–46].

## Differences between semi-rigid (terrestrial) and highly flexible (aquatic) vegetation

In the previous section we summarised the influence of vegetation on flow from theoretical work and observations both in terrestrial and aquatic environments. The majority of aquatic canopy layer studies have used vegetation analogous in morphology and biomechanical properties to that used within the terrestrial environment [5, 47] or have focussed on aquatic equivalents such as seagrasses [7]. However, aquatic vegetation in rivers exhibits a wide range of forms and can be significantly different to terrestrial vegetation in morphology and dynamical behaviour. Here we suggest that there are three main considerations which must be taken into account when comparing highly flexible aquatic canopies with their terrestrial counterparts.

### Depth-limitation of aquatic flows

Within terrestrial canopies, where the canopy height is small in comparison to the boundary layer height, canopy mixing layer processes interact with the larger scale boundary layer hairpin vortices [17]. Contrastingly, aquatic flows are depth-limited and therefore boundary layer development is restricted and the flow may be dominated by the K-H instability process in the mixing layer [6, 48]. Furthermore, vegetation growth is depth-limited through light availability, and therefore deeper aquatic flows where boundary layers may be more significant are less likely to be heavily vegetated [49–51].

### Biomechanical properties and force balance

Within terrestrial environments, plants rely upon rigidity to support their own weight as they grow to compete for light [52]. Conversely, within aquatic environments where the fluid density is 1000 times greater and therefore the density difference between the plant and the fluid is smaller, rigidity is less important, allowing aquatic plants to be more flexible [53]. Furthermore, aquatic species can be positively buoyant [54] and therefore do not rely upon rigidity to compete for light. While rigidity can still be important, particularly for emergent aquatic plants (e.g. *Phragmites* spp.), the majority of macrophytes exhibit low flexural rigidity in response to drag [19, 54]. Aquatic plants can experience a drag force 25 times larger than terrestrial plants for a given velocity [51, 55]. Therefore, low rigidity enables aquatic plants to reconfigure within the flow to minimize the drag and prevent uprooting or damage [56].

The differences between the terrestrial and aquatic environments create different force balances. In the semi-rigid terrestrial case, the main forces acting on the stem are the drag (*F*_*D*_) and the internal rigidity force (*F*_*R*_), whereas in the highly flexible aquatic case, the main forces are the drag force and the buoyancy force (*F*_*B*_). These two types of plant may be characterised broadly as ‘bending’ and ‘tensile’ plants [57]. This classification is made on the basis of the Cauchy number (*Ca*) which is the balance between the drag force and the rigidity force.

1 $$Ca = F_{D} /F_{R}$$

Nikora [57] categorised plants with large values of *Ca* as tensile plants and those with small values of *Ca* as bending plants. Luhar and Nepf [54] extended this approach by characterising the spectrum of vegetation behaviour using both the Cauchy and the Buoyancy number (B).

2 $$B = F_{B} /F_{R}$$

They used these two parameters and their ratio, which between them represent the ratios between the three key forces, to predict plant reconfiguration. The classification of plant (i.e. bending or tensile) will have an impact upon plant-flow interactions, such as flow modulation by the natural frequency of the vegetation which is likely to be more prevalent in bending canopies.

### Posture and form

As a result of the different force balance, many aquatic plants adopt a horizontal position within the flow, which is a departure from the idealized, perpendicular canopy structure used within terrestrial canopies and many aquatic prototype experiments [47, 58]. It is therefore likely that plant-flow interactions will reflect that. Aquatic vegetation must find a balance between drag reduction and photosynthetic capacity [59, 60]. Therefore, aquatic vegetation commonly has substantial foliage with a large surface area to maximize light capture. As a result, aquatic vegetation is often characterized by complex plant morphology, which the canopy mixing layer model does not account for. This may be significant in terms of flow structure as foliage can inhibit momentum exchange between the canopy flow and the flow above [61].

Considering all these factors, flow structure and flow–vegetation interaction within aquatic canopies may be potentially quite different to terrestrial counterparts. However, our theoretical understanding of aquatic vegetation is still firmly based on our process understanding of semi-rigid terrestrial vegetation. Simulating flow through both semi-rigid and highly flexible canopies enables us to assess whether using the theoretical framework generated from work in terrestrial canopies is directly transferable to aquatic canopies.

## Methods

### Design of experiments

**Fig. 2:**
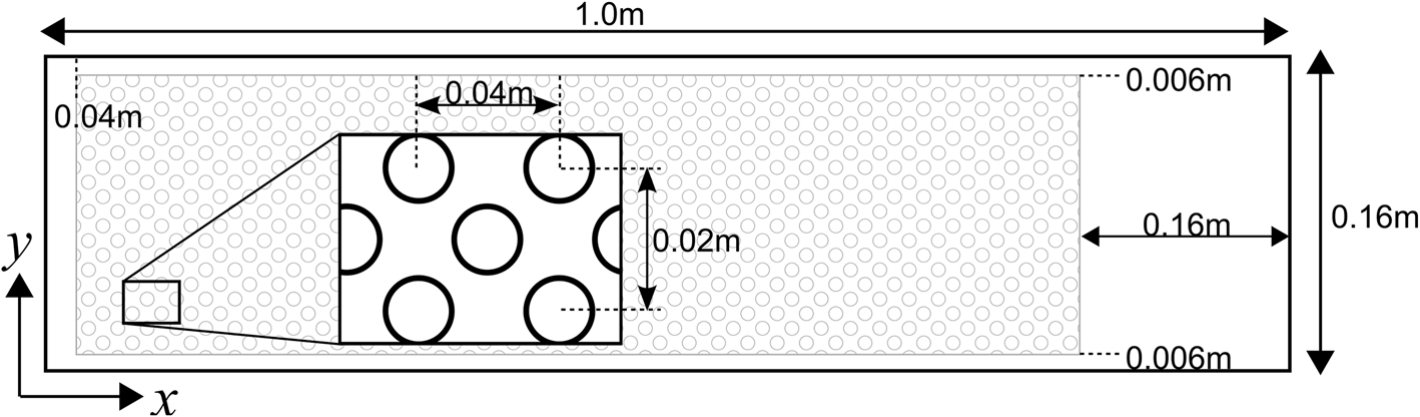
Plan view schematic of the simulation setup with flow from *left to right* with the vegetation canopy shown by the *shaded region*. Domain not drawn to scale

In order to simulate flow over a canopy, numerical simulations were conducted using a domain 1 m long (*l*), 0.16 m wide (*b*) and 0.32 m deep (*h*) (Fig. 2). A canopy of 300 stems was placed within the domain, with a solid volume fraction of $\phi = 0.176$ (frontal area per canopy volume, *a* = 25 m^−1^) which represents dense aquatic vegetation and is of a similar order to that used in other canopy studies [62]. Each stem was 0.15 m tall with a radius of 0.005 m, a material density of 950 kg m^−3^ and a flexural rigidity of 3.0 × 10^−4^ Nm^2^ for the semi-rigid case (*Ca* ≈ 5, *B* ≈ 0.40) and 3.0 × 10^−8^ Nm^2^ for the highly flexible case (*Ca* ≈ 50,000, *B* ≈ 4000). The stems were positioned in a staggered arrangement (Fig. 2). The bed was simulated using a no-slip condition and a logarithmic wall function (*y* ^+^ ≈ 20–40) while, the sidewalls of the domain were simulated as frictionless boundaries to minimise domain-induced wall effects. The free surface was simulated using a rigid-lid treatment. A periodic boundary condition was used at the inlet to allow the full development of a canopy layer profile with a mean domain velocity of 0.3 ms^−1^. The flow was fully turbulent and sub-critical. Flow was simulated for 60 s, of which the final 30 s of data (approximately 9 flow-throughs) were recorded for analysis.

### Numerical solver

The numerical experiments were conducted within a three-dimensional computational fluid dynamics (CFD) framework within which the Navier–Stokes equations for mass and momentum were coupled and solved using the SIMPLEST algorithm [63]. In this algorithm, an initial pressure field is prescribed which is then used to solve the momentum equations. A pressure correction equation is then applied to ensure continuity. This updated pressure field is then used to solve the momentum equations again and this iterative process is repeated until residual errors are reduced to 0.1 % of the inlet flux. A regular Cartesian grid with cell size of 0.002 m in each direction was used and the flow was solved using staggered grids for scalar and vector variables. In order to balance the demands of accuracy and stability, a second order, bounded, upwind differencing scheme was used for the convective terms, while central differencing was used for the diffusive terms. The Navier–Stokes equations were solved using Large Eddy Simulation (LES), with a constant Smagorinsky sub-grid scale model (*C*_*S*_ = 0.17). The vegetation stems were represented as an immersed boundary within the domain using a dynamic mass flux scaling algorithm [64], whereby individual cell porosities are altered to account for the presence of dynamic mass blockages within the flow without the need for adaptive re-meshing at each time-step [20]. Therefore, in contrast to many LES studies which use fitted grids, with refinement near boundaries, this method represents a low-resolution LES approach, similar to that of Kim and Stoesser [65]. Consequently, fine-scale turbulent vortices shed from the individual stems into the wake are not resolved within the model. The impact of this simplification is discussed in Sect. 5.2. The fluid–structure interaction was solved in a sequentially staggered manner [66], such that velocity and pressure data were passed from the fluid model after each time-step in order to derive plant motion and then new plant position data were fed back into the fluid model for the next time-step. The drag force provided the coupling between the flow and plant models, while other fluid forces where not considered for simplicity. Thus, the effect of the vegetation on flow was incorporated directly through the mass blockage, no slip boundary condition at blocked cell edges and resulting drag force. The corresponding fluid drag force acting on the stems was then calculated from the LES pressure and velocity data interpolated at the stem boundary. The plant position was then solved by balancing the external drag force against the internal inertial and bending stiffness forces [20].

### Biomechanical models

To simulate plant motion, two different biomechanical models were applied. These two models were used to represent the two different vegetation types described in Sect. 3.2. The first was based upon the Euler–Bernoulli beam equation and is applicable to semi-rigid, ‘bending’ vegetation (*Ca* ≈ *O*(1), *B* < *O*(1)). Each stem is represented as a cantilever beam and shear effects are neglected. This type of model has previously been successfully applied to semi-rigid vegetation canopies [67, 68]. The second model is based on an N-pendula approach and treats each vegetation stem as a series of pendula connected by “hinges” or “joints”. This model is suitable for modelling highly flexible ‘tensile’ vegetation (*Ca* ≫ 1, *B* ≫ *O*(1)) with low rigidity and localised bending. Similar models have previously been applied to seagrasses [19, 69]. Full details concerning the two biomechanical models are reported by Marjoribanks et al. [20].

### Analysis methods

In order to compare the results within the canopy mixing layer theory framework, four main analysis methods, which have been used previously to characterise canopy mixing layers [7, 8, 17, 32, 70] are applied to the data.

#### Normalised velocity and Reynolds stress profiles

These are calculated using temporally averaged flow data extracted from the end of the canopy, spatially averaged across the canopy width (*x*/*l* = 0.84). The variables are normalised following the approach of Ghisalberti and Nepf [7]. In these equations, *U* and $\overline{{u^{{\prime }} w^{{\prime }} }}$ are both temporally averaged but are functions of height (*z*), $\bar{U}$ is defined as the arithmetic mean velocity of the two flow regions, Δ*U* is the difference between the mean velocities within the two flow regions, *θ* is the momentum thickness which is a measure of the thickness of the shear layer, and $\bar{z}$ is defined such that $U(\bar{z}) = \bar{U}$. These normalised velocity profiles allow comparison of the data to a conventional mixing layer and can also be used to calculate key mixing layer variables such as the mixing-layer induced KH vortex frequency (*f*_*KH*_) [7, 31].

3 $$U^{\ast} = \frac{{U - \bar{U}}}{{{{\Updelta}}U}}$$

4 $$\overline{{u^{{\prime }} w^{{\prime }} }}^{\ast} = \frac{{\overline{{u^{{\prime }} w^{{\prime }} }} }}{{{{\Updelta}}U^{2} }}$$

5 $$\theta = \mathop \int \limits_{ - \infty }^{\infty } \left[ {\frac{1}{4} - \left( {\frac{{U - \bar{U}}}{{{{\Updelta}}U}}} \right)^{2} } \right]dz$$

6 $$z^{\ast} = \frac{{z - \bar{z}}}{{\theta_{M} }}$$

7 $$f_{KH} = 0.032\frac{{\bar{U}}}{\theta }$$

The mixing layer velocity profiles are compared to the typical hyperbolic tangent profile of a mixing layer [7]. The Reynolds stress profiles are compared to two previous studies. Firstly, the profile of Rogers and Moser [71], who used direct numerical simulation (DNS) to study plane mixing layers, is used as a comparison to a classical mixing layer theory. Secondly, the results are compared to the theoretical profile developed by Sukhodolov and Sukhodolova [72] for vegetated mixing layers using scaling laws and the turbulent viscosity model.

#### Spectral and wavelet analysis

Time series analysis using both a Fourier and wavelet transform is applied for the full duration of the measurement period at a point along the centre line of the domain (*y*/*b* = 0.5) at the downstream end of the canopy (*x*/*l* = 0.84) just above the canopy-top to ensure no interference from stems (*z*/*h* = 0.5). This enables the identification of key periodicities within the flow and is therefore used for assessing the representation of turbulence within the LES model and comparing observed vortex frequencies with those predicted using the canopy mixing layer model (Eq. 7). A key advantage of wavelet analysis over other frequency transformations such as spectral analysis is that it retains a temporal dimension which shows how periodicities change through time [73]. The Morlet wavelet is fitted to the data across scales from 0.04 s to 20.48 s, centred at each point in the time series to calculate the wavelet power spectrum. Points that do not have statistically significant wavelet power compared to a white noise spectrum, and those subject to edge effects are discarded and the wavelet scale is converted to the equivalent Fourier period for comparison with other data [20, 74]. For the power spectral analysis, the Welch periodogram method was applied to the time series data, with two non-overlapping windows [75].

#### Quadrant analysis

Quadrant analysis is applied to identify the presence of sweep and ejection events within the flow [34]. Here, downstream (*u*) and vertical velocity (*w*) time series extracted from an *x*–*z* plane along the midline of the domain (*y*/*b* = 0.5) are decomposed into mean and fluctuating components using Reynolds decomposition. The fluctuating velocities are then plotted onto a quadrant plot which divides the flow into a series of 4 distinct quadrant events: outward interactions, ejections, inward interactions and sweeps [34]. In order to exclude low energy, small-scale fluctuations, a hole-size (H) condition is applied which excludes data where $\left| {u^{{\prime }} w^{{\prime }} } \right| < Hu_{RMS} w_{RMS}$ with a hole size of *H* = 2 [34].

#### Eulerian and Lagrangian vortex detection methods

To investigate the presence and nature of vortices within the flow, both Eulerian and Lagrangian vortex detection methods are applied. For the Eulerian methods, the Q criterion [76] is used which identifies regions where the magnitude of the vorticity vector is greater than that of the rate of strain. In order to determine the distribution of vortex size, the size of every vortex identified by the Q criterion was measured for an *x*–z slice down the centre-line of the domain for all time-steps. Only the data above the mean canopy top were used to avoid capturing small-scale and fragmented vortices within the canopy. In addition to the Q criterion, the spanwise component of the vorticity vector is presented, which provides a less stringent condition on vorticity as it is unable to determine between regions of high lateral shear and vorticity [77] but does retain information on the directionality of the vortices. Finally, the Lagrangian analysis applied the Finite-time Lyapunov exponent (FTLE) method, which tracks individual fluid trajectories back through time to identify regions of attracting phase-space [78, 79]. This method is limited by fluid trajectories tracking back upstream of the domain inlet, and therefore the time period for tracking trajectories must balance the benefits of increased tracking back period [80] against the size of the region of the domain for which a full trajectory can be calculated. In this case, a track-back period of 0.5 s was applied and regions near the inlet without valid trajectories are shown as no data. Vortices are identified as regions of attracting flow with ridges in the FTLE field highlighting the presence of Lagrangian coherent structures [80].

## Results

### Description of the flow and normalised flow profiles

**Fig. 3:**
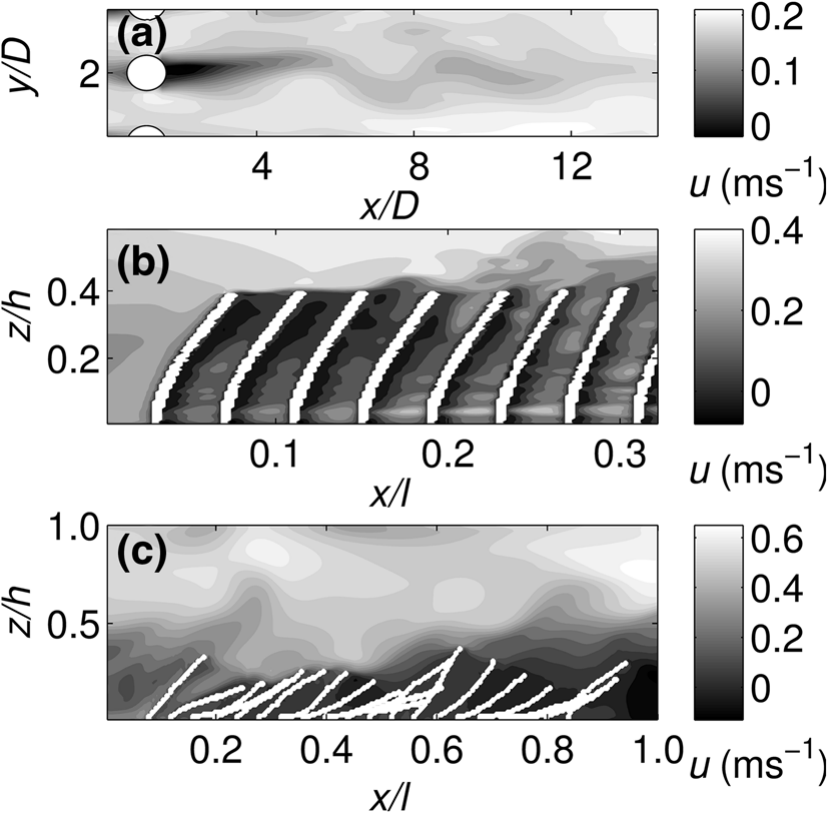
Instantaneous snapshots of **a** wake flow, **b** shear flow and **c** the entire domain. **b**, **c** demonstrate typical plant positions for the semi-rigid and highly-flexible canopies respectively. Flow is from *left to right*

Instantaneous snapshots of the velocity field (Fig. 3) demonstrate that the model captures both stem-scale and canopy shear layer scale flow processes. At the stem-scale (Fig. 3a) there is evidence of individual unstable stem wakes leading to the formation of a vortex street. Stem Reynolds number values vary between *Re* ≈ 300–2000 along the stem depending on the local velocity. For the semi-rigid canopy (Fig. 3b), the flow quickly develops into a typical canopy shear layer characterised by a sharp velocity gradient at the canopy top, and formation of coherent turbulent structures along the canopy top. For the highly flexible canopy, this shear layer is less well defined and there is evidence of more complex flow structure due to the more prone position of the vegetation and increased plant motion (Fig. 3c). For example, the canopy height is much more varied than in the semi-rigid case exhibiting large scale streamwise undulations.

**Fig. 4:**
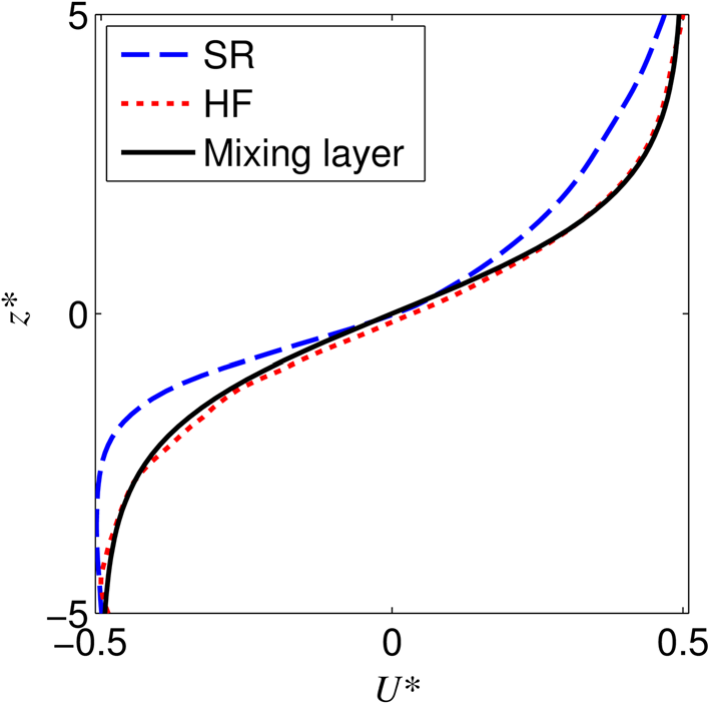
Normalised velocity profiles for the semi-rigid (SR) and highly flexible (HF) canopies, as well as the idealised mixing layer profile as used by Ghisalberti and Nepf [7]

**Fig. 5:**
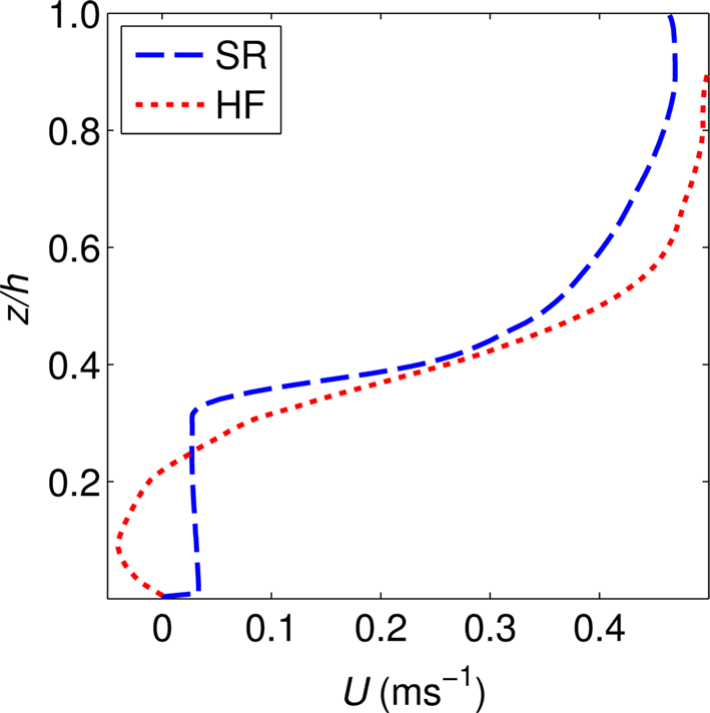
Downstream velocity profiles for the semi-rigid (SR) and highly flexible (HF) canopies

The normalised velocity profiles (Fig. 4) show that for both the semi-rigid (SR) and highly flexible (HF) canopies the flow is well described by a mixing layer. This is particularly the case for the highly flexible case which maps closely onto the idealised mixing layer profile. The semi-rigid case shows substantial asymmetry about the centre of the mixing layer with a steep decrease in velocity towards the canopy region (z* < 0). The momentum thickness of the shear layers (θ, Eq. 5), calculated from the normalised profiles is 0.021 m for the highly flexible case and 0.016 m for the semi-rigid case. This suggests that for the highly flexible case the shear layer is thicker. The normalised variables estimate the KH vortex frequencies (Eq. 6) for the semi-rigid and highly flexible canopies as 0.52 and 0.42 Hz respectively. While the normalised profiles characterise the flow over the mixing layer regions they do not provide information on the location or dimensional width of the mixing layer. Therefore, the dimensional velocity profiles are also considered (Fig. 5). These profiles show the difference between the two cases with a much wider and lower gradient shear layer in the highly flexible canopy case, as compared with the asymmetric, narrow and high velocity gradient mixing layer evident within the semi-rigid case. This highlights the generalising effect of the normalisation process which can remove significant differences in the velocity profiles and is not a sensitive indicator of self-similarity [71].

**Fig. 6:**
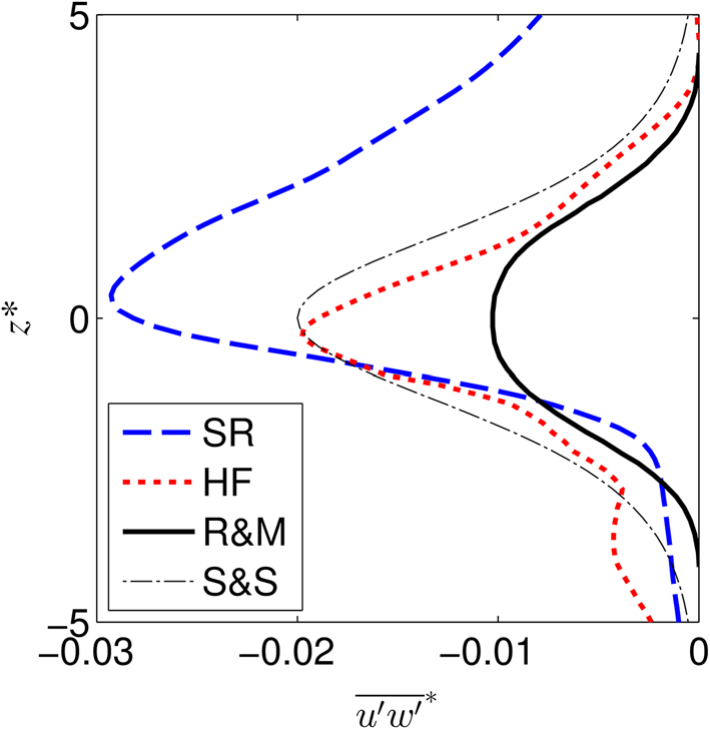
Normalised Reynolds stress profiles for the semi-rigid (SR) and highly flexible (HF) canopies. The experimental mixing layer profile of Rogers and Moser [71] (R&M) and the theoretical canopy profile of Sukhodolov and Sukhodolova [72] (S&S) are also shown

The normalised Reynolds stress profiles (Fig. 6) provide a more sensitive indicator and show that both the highly flexible and semi-rigid cases have Reynolds stress peaks larger than those typical of a classical mixing layer [71]. The highly flexible profile is similar in shape and magnitude to the theoretical profile derived by Sukhodolov and Sukhodolova [72] (*γ* = 0.02) for vegetated mixing layers which also agreed well with their field data. The highly flexible profile also displays a smaller secondary peak below the centre of the mixing layer (z* ≈ −4), which may indicate the presence of additional turbulent processes within the canopy due to either plant motion or flow recirculation within the canopy. This secondary peak is ≈20 % of the mixing layer peak magnitude and is not present within the semi-rigid case. A similar peak is seen in the data of Okamoto and Nezu [8] for a canopy exhibiting *monami*. The semi-rigid profile confirms the asymmetry evident in the velocity profile, with a much steeper decrease in Reynolds stress towards the canopy (z* < 0). The magnitude of the Reynolds stress peak is 50 % higher than the highly flexible case and over 200 % higher than the classical mixing layer case. This is due in part to the increased velocity difference (Δ*U*) in the highly flexible canopy, as shown in Fig. 5 which in turn decreases the normalised Reynolds stress (Eq. 4).

### Spectral and wavelet analysis

**Fig. 7:**
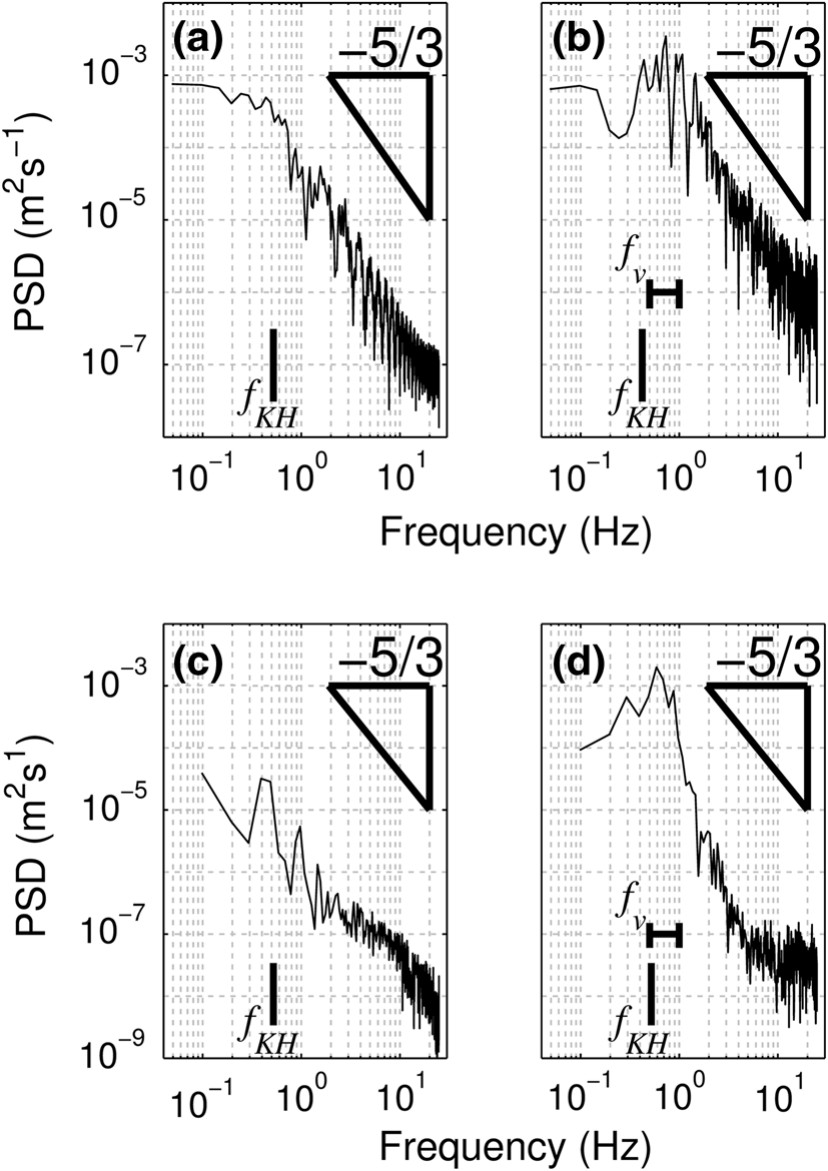
Power spectra for the velocity (**a**, **b**) and stem height (**c**, **d**) time series for the semi-rigid (**a**, **c**) and highly flexible (**b**, **d**) canopies. The Kolmogorov −5/3 scale is shown by the *triangle* while the *lines* represent the scales corresponding to the predicted K–H (*f*_*KH*_) and vegetation-induced (*f*_*v*_) frequencies

The velocity power spectra for both simulations (Fig. 7a, b) indicate that the turbulence predominantly follows the expected Kolmogorov decay rate, indicating that all the scales of interest lie within the inertial subrange and that the model accurately reproduces the turbulent processes with this range, with minimal impact of numerical diffusion or energy dissipation due to the SGS model [81, 82]. As discussed in Sect. 4.2, fine-scale turbulence at the plant wake-scale is not resolved by the model and therefore experimental data are required to verify the model’s performance at such scales where, in similar models, low grid resolution has been shown to result in under-prediction of Reynolds stresses [83]. At larger scales, both flow spectra exhibit peaks close to the predicted KH frequencies (as labelled in Fig. 7). In the semi-rigid case, this is a single, well-defined peak. In contrast, for the highly flexible canopy, there is a broader peak, which extends to higher frequencies beyond the predicted KH frequency. The plant motion spectra both display similar peaks to the flow spectra highlighting the coherence between flow and plant motion.

**Fig. 8:**
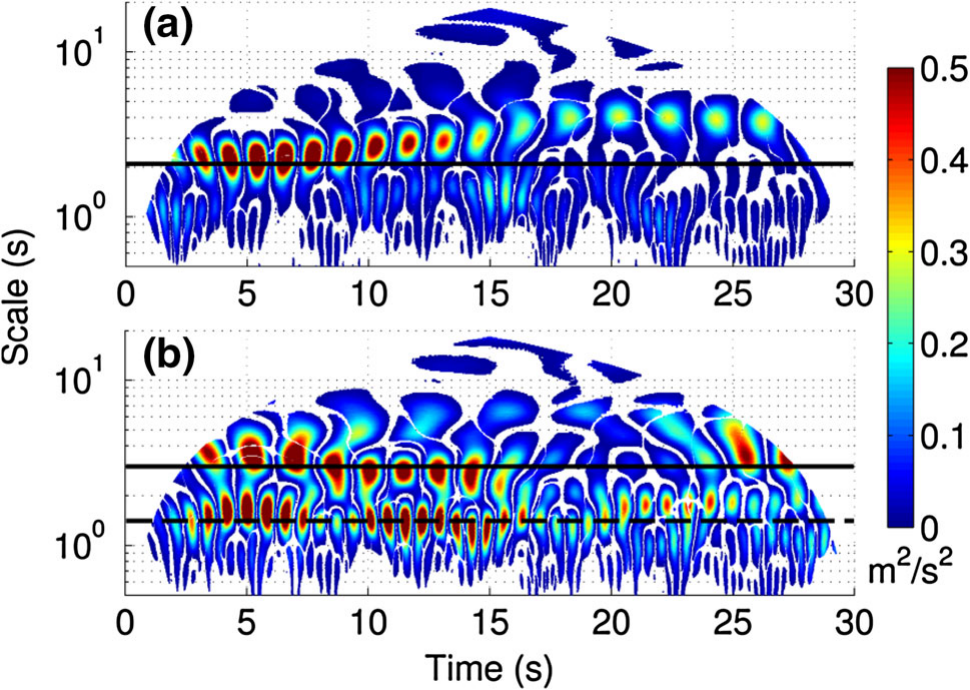
Wavelet spectra for the semi-rigid (**a**) and highly flexible (**b**) canopies. The *black lines* indicate the predicted KH vortex frequencies

The wavelet plot for the semi-rigid canopy (Fig. 8a) shows a similar pattern to the spectral analysis, with a single dominant periodicity which is initially at the KH frequency predicted from the normalised profiles (*f*_*KH*_ = 0.52, scale = 1.92 s, shown by black line in Fig. 8a) but then decreases in frequency and wavelet power in the second half of the simulation. This suggests that local canopy variables may cause the frequency to fluctuate through time. The dominance of the single mixing layer scale periodicity implies that the turbulence regime is controlled by the mixing layer. In contrast, the highly flexible wavelet plot (Fig. 8b) shows a larger range of concurrent scales of periodicity as shown by the velocity spectra. There is a clear periodicity at the predicted KH frequency (*f*_*KH*_ = 0.42 Hz, scale = 2.38 s), which as with the semi-rigid case appears to vary through time and is less well defined than in the semi-rigid case. At approximately 15 s this periodicity appears to decrease in power and potentially merge with the higher frequency scale before reappearing towards the end of the simulation. There is also a distinct lower scale (higher frequency) periodicity between 1 and 2 s (0.5–1 Hz) (Fig. 8b, dotted line). This signal suggests the presence of additional turbulent processes within the canopy mixing layer region, possibly linked to the secondary peak in the Reynolds stress profile. This scale is greater than that predicted for stem-wake generated turbulence at the canopy top (*f*_*W*_ = 0.2*U*/*D* ≈ 6) and therefore we suggest that this turbulence may relate to plant motion processes. This higher frequency signal contains significant energy with a similar magnitude wavelet power to the mixing layer periodicity, suggesting it contributes substantially to the overall TKE budget. Similar to the lower frequency periodicity, it also shows significant variation in frequency over the duration of simulation. This periodicity agrees well with the velocity power spectra (*f*_*V*_ in Fig. 7b) where the turbulence production range extends to frequencies beyond the predicted KH frequency. There is also evidence of a lower frequency, lower power periodicity, which appears to separate from the mixing layer frequency temporarily between 10 s and 25 s.

### Quadrant analysis

**Fig. 9:**
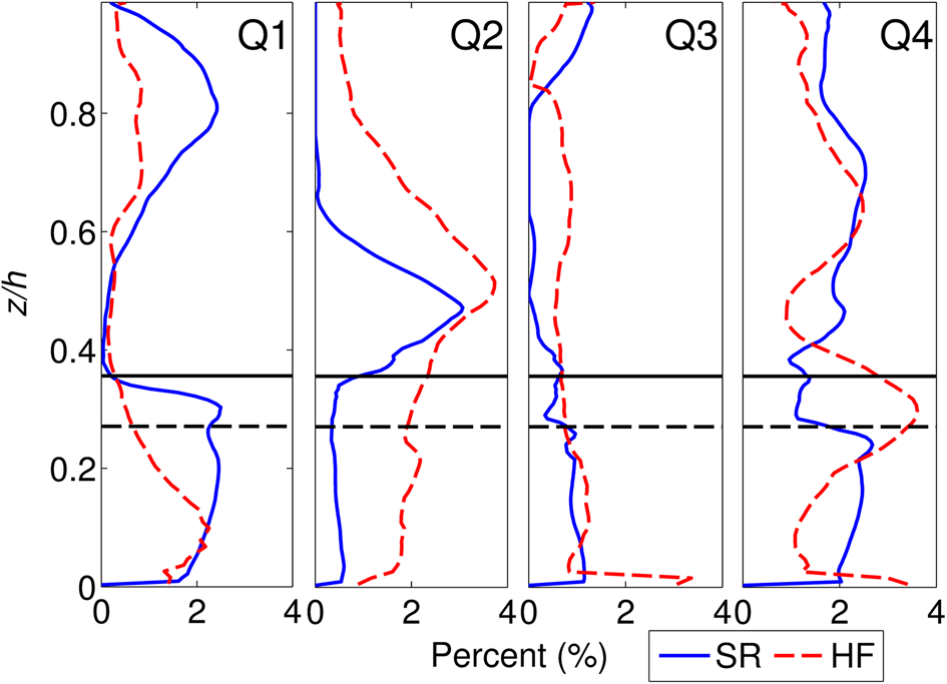
Quadrant profiles for the semi-rigid (SR) and highly flexible (HF) canopies showing the vertical distribution of high energy quadrant events (H = 2). Approximate canopy heights are shown by the *black lines* for the SR (*solid*) and HF (*dashed*) cases

The distribution of high magnitude quadrant events (Fig. 9) shows a dominance of sweeps (Q4) within the canopy and a stronger dominance of ejection events above the canopy for both the semi-rigid and highly flexible cases. Within each case, the peak values for sweeps and ejections are similar, with the highly flexible canopy exhibiting a 20–30 % increase in occurrence of both. There is also a small peak in sweep events above the mixing layer in both cases. The sweep profiles are similar throughout the flow depth, although the highly flexible case has a higher proportion of sweep events at the top of the canopy (the pattern is reversed for the lower canopy). In contrast, the ejection profiles are less similar, with a larger ‘background’ level of ejection events in the highly flexible canopy, approximately 1–2 % higher occurrence than for the semi-rigid case, which extends throughout the flow depth.

Inward interactions (Q3) show very little variation with height, with a relatively consistent low level (1 %) throughout the flow depth, suggesting that the canopy flow regime has very little impact upon these events. Outward interactions (Q1) are prevalent within the canopy for both cases. This has been found in previous studies [36] and attributed to the impact of vegetation motion and the impact of a few large magnitude events penetrating into the low velocity region within the canopy. However, other studies have found no evidence of such a peak in outward interactions [84] and while this may be due to differences in flexibility or in stem density between cases, this remains an area for further work. The contributions of outward and inward interactions diminish towards the canopy top, suggesting increased coherence within the mixing layer [23]. Similar to the sweeps, there appears to be a secondary peak above the mixing layer though the cause of this is unknown.

### Vortex detection methods

**Fig. 10:**
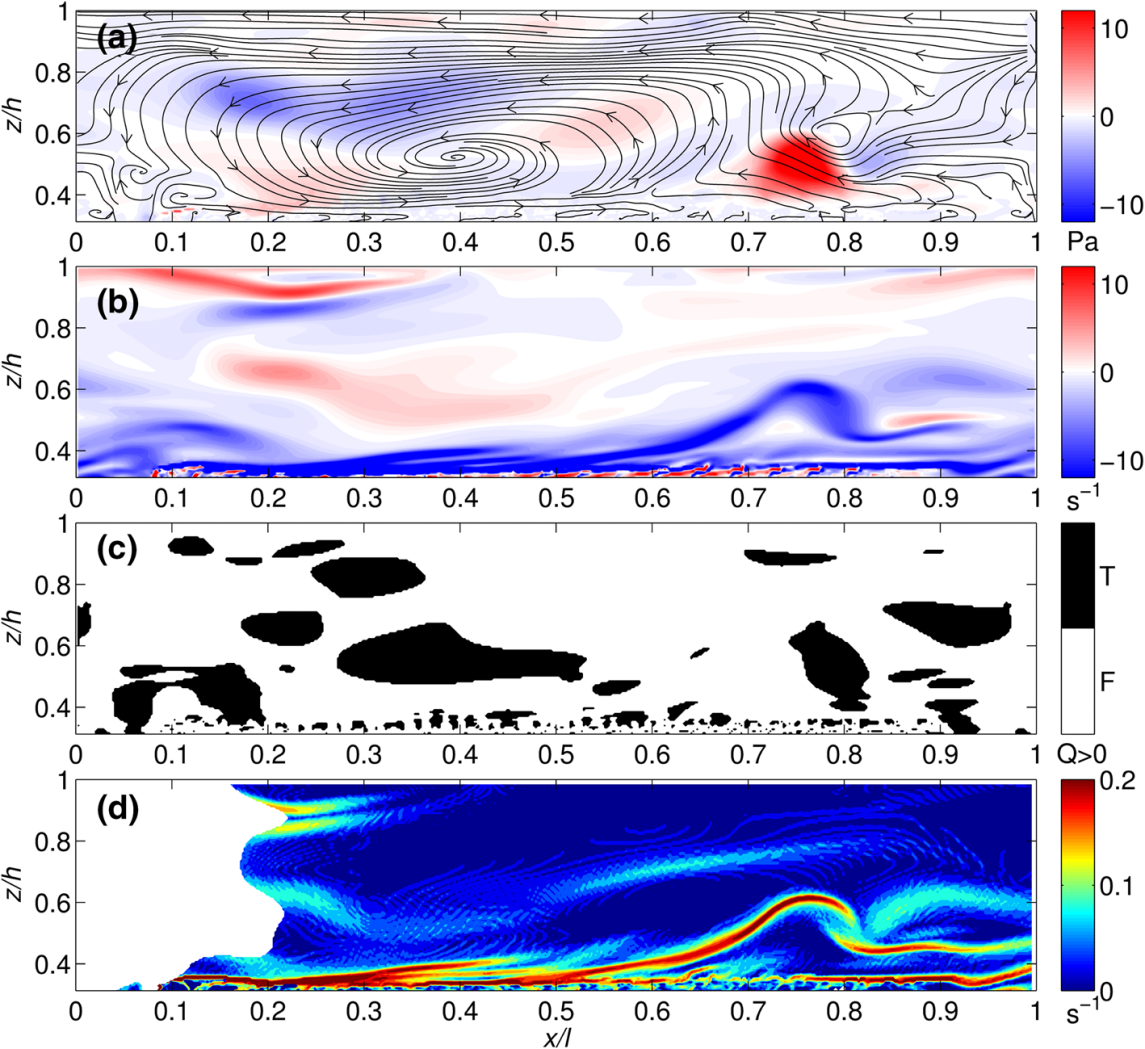
Vortex identification for the semi-rigid canopy using **a** Reynolds stress (*contours*) and instantaneous velocities (*streamlines*), **b** vorticity, **c** Q criterion and **d** FTLE methods. Flow is from *left to right* and for clarity, only flow above the canopy is shown. The mean canopy height is at 0.35*z*/*h*

**Fig. 11:**
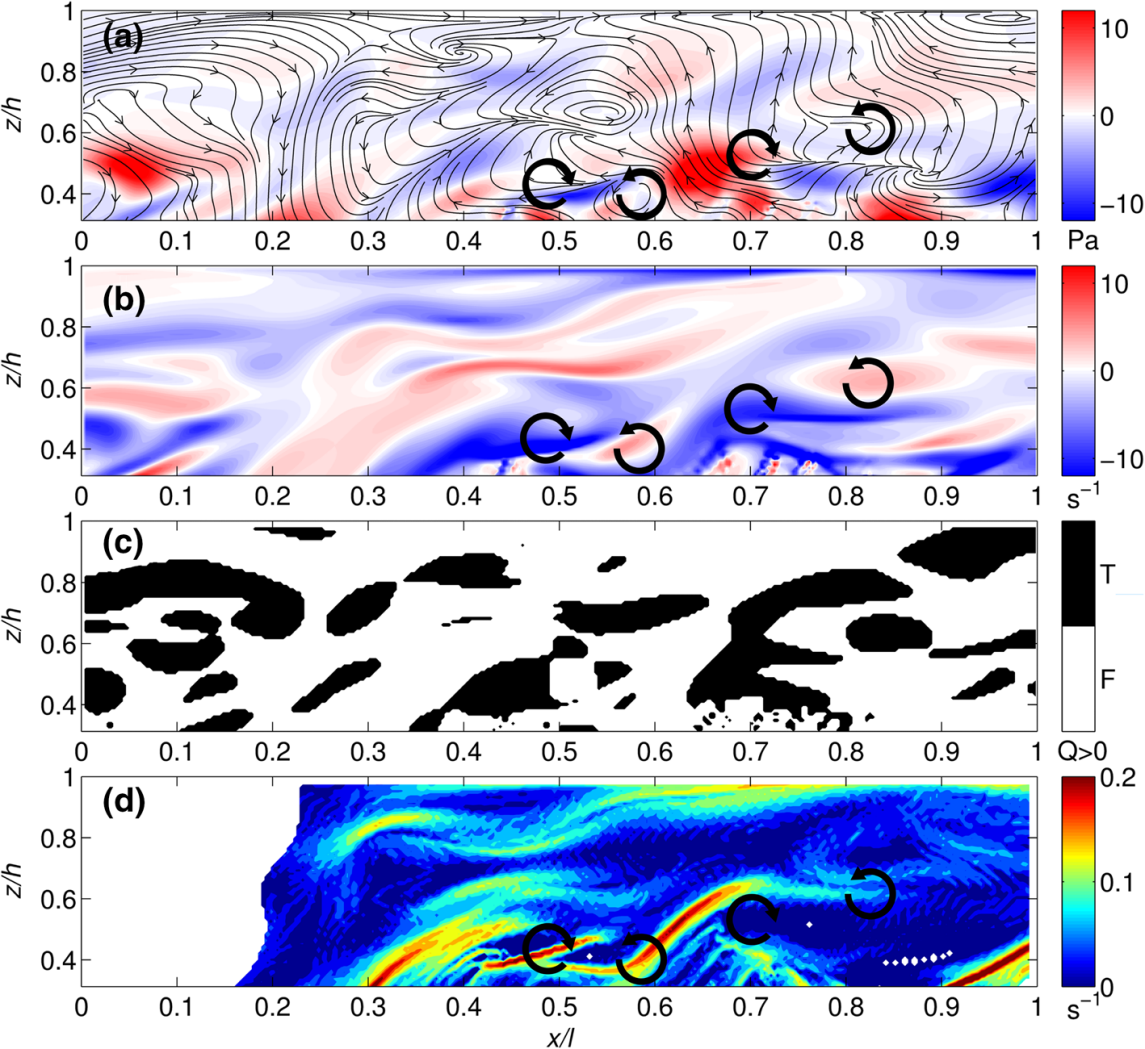
Vortex identification for the highly flexible canopy using **a** Reynolds stress (*contours*) and instantaneous velocities (*streamlines*), **b** vorticity, **c** Q criterion and **d** FTLE methods. Flow is from *left to right* and for clarity, only flow above the canopy is shown. *Black arrows* highlight the presence of potentially plant-shed vortices. The mean canopy height is at 0.27*z*/*h*

The snapshots of velocity and vorticity within the flow (Figs. 10 and 11) provide insight into the instantaneous vorticity field. For the semi-rigid canopy case (Fig. 10), the instantaneous velocity streamlines (Fig. 10a) highlight the presence of the large-scale coherent structures within the flow. The highest magnitude Reynolds stresses correspond to a structure just above the canopy top (*z*/*h* ~ 0.5) at approximately *x*/*l* = 0.8. The vorticity field (Fig. 10b) shows the dominance of clockwise (negative) vorticity concentrated along the canopy top and identifies the structure at *x*/*l* = 0.8 as a clockwise vortex, consistent with a mixing layer roller or possibly hairpin vortex. Above the canopy there are weaker, large-scale vortices which appear stretched in the downstream direction, including the structure identified by the velocity streamlines in Fig. 10a, centred at *x*/*l* = 0.4. The Q criterion (Fig. 10c) supports these findings, identifying a small number of large-scale vortices as well as much smaller scale vortices at the canopy top. The FTLE ridges (Fig. 10d) also highlight the canopy top as the main region of vorticity, with the clear formation of a roller vortex at the canopy [78]. Marjoribanks et al. [20] demonstrated that the growth rate of this roller vortex is consistent with that associated with mixing layer growth.

The velocity and vorticity plots for the highly flexible canopy (Fig. 11a, b) show a more complex distribution of vorticity which extends throughout the full depth of the flow and includes substantial additional regions of anti-clockwise vorticity. Over the duration of the simulation, 64 % of the above-canopy domain exhibits positive, anti-clockwise vorticity, in comparison to 41 % for the semi-rigid case. There is also evidence of potential vortex shedding from individual stems (as labelled by the arrows in Fig. 11). The Reynolds stress patterns (Fig. 11a) show greater magnitudes of Reynolds stress within the highly flexible canopy, as compared with the semi-rigid canopy. This appears in contrast to the Reynolds stress profiles (Fig. 7). However, as discussed earlier, the normalised Reynolds stress values are scaled by the velocity difference of the shear layer. Therefore, Fig. 11a demonstrates that there are high values of Reynolds stress within the flow, but these do not relate to the strength of the shear layer (i.e. they are the result of additional turbulent processes). The Q criterion (Fig. 11c) identifies a larger coverage of vortices than in the semi-rigid canopy, and the individual vortices are visually more complex in form. The FTLE results (Fig. 11d) highlight vortex ridges extending from the canopy top into the main flow. The pattern is more complex than the semi-rigid case, with more vortex ridges present. The FTLE field also highlights the ridge between counter-rotating vortices which appear to be shed alternately from the canopy top at this instant.

**Fig. 12:**
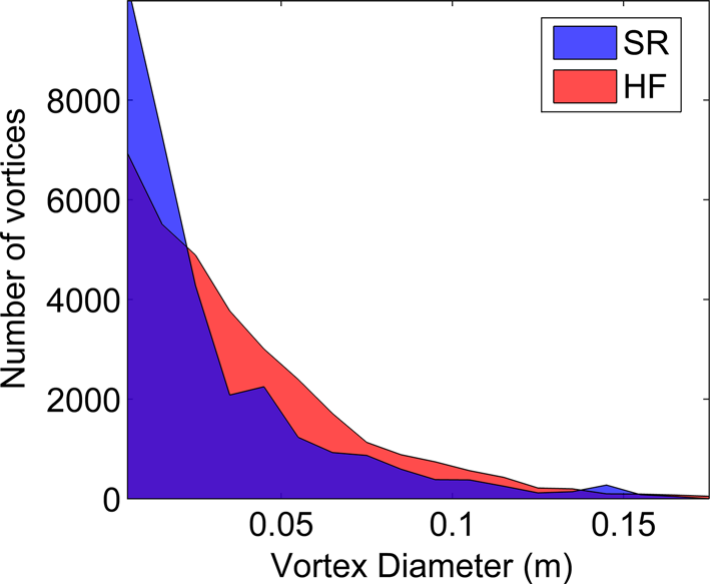
Occurrence of different sized vortices throughout a 2D *x*–*z* slice of the domain for the duration of the simulation for the semi-rigid (SR) and highly flexible (HF) canopies

In order to assess whether these observations generalise throughout the simulation, the vortex size distribution over the entire simulation is assessed statistically. This was calculated by measuring the maximum width in the vertical (*z*) direction of each vortex at each time-step throughout the duration of the simulation for an *x*–*z* slice along the centreline of the model domain. The resulting distribution of vortex diameters (Fig. 12), shows that the two cases are broadly similar with an increasing occurrence of vortices with decreasing size, which is expected given turbulence decay processes. The integral length-scale associated with the depth of the flow is 0.32 m, however the dense canopy and high shear means that such vortices are unlikely to remain intact. Instead, the integral vortex size scales with the open flow above the canopy (~0.17 m). This is demonstrated clearly in Fig. 12. The average number of vortices observed at each time-step is similar (SR = 21.1, HF = 21.81). However, there are noticeable differences in the distribution of vortex size that suggest different turbulent production mechanisms between the flows, occurring at a range of scales. Primarily, the semi-rigid canopy produces more small-scale (<0.02 m) vortices whereas the highly flexible canopy produces more mid-scale vortices (0.02–0.1 m). For the largest vortices (>0.1 m) the distribution is similar between the two cases, with only minor differences. These three regions can be broadly related to different turbulent mechanisms within the flow.

**Fig. 13:**
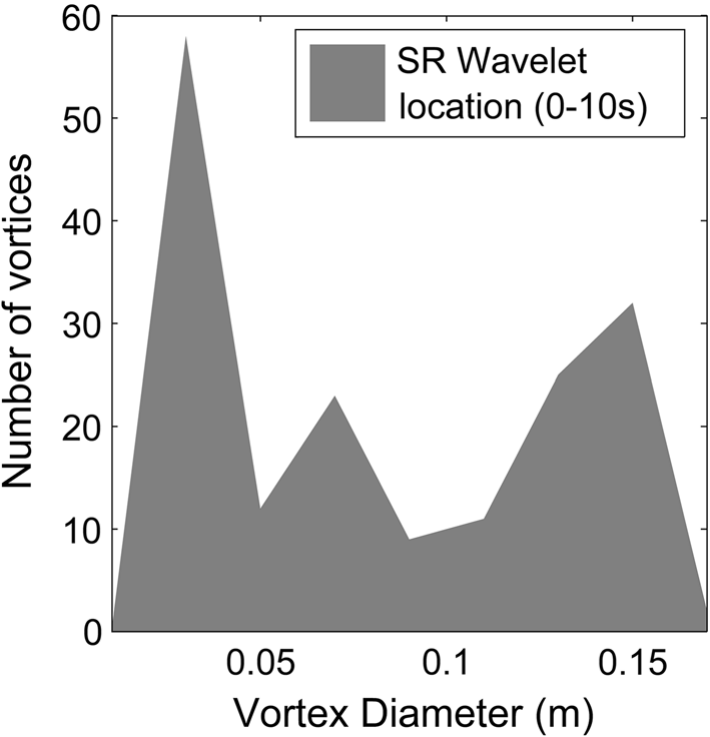
Occurrence of different sized vortices at the location of the time series extracted for the wavelet analysis during the first 10 s of the semi-rigid canopy simulation

Firstly, the largest vortices (>0.1 m) correspond to shear layer vortices. This can be seen by examining the distribution of vortex diameter of vortices crossing the location of the time series extracted for the wavelet analysis. For the first 10 s of the semi-rigid canopy measurement period, the wavelet spectra (Fig. 8a) are dominated by a single low frequency periodicity. The distribution of vortex size at the time series location for this period (Fig. 13) shows that this larger scale vorticity most likely corresponds to the peak in vortex size between 0.10 and 0.15 m. This is supported by the data of Marjoribanks et al. [20] who measured a shear-layer generated vortex reaching a width of 0.1 m by the end of the canopy. Secondly, we suggest that the difference in distribution of small-scale vortices (<0.02 m) relates to additional stem-wake generated vortices. These can be identified in Fig. 11b at the canopy top. Assuming Taylor’s frozen turbulence hypothesis holds for these small scale vortices, a vortex diameter of 0.02 m represents a frequency of approximately 6.25 Hz which is consistent with that predicted for the wake shedding mechanism at the canopy top.

**Fig. 14:**
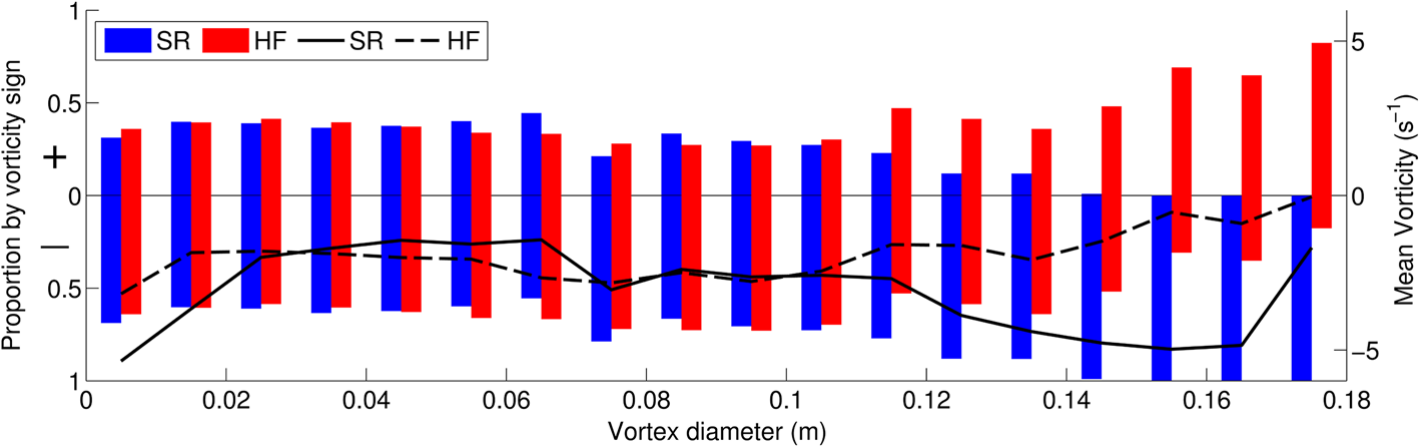
Distribution of vortex sign (rotation direction) and mean vorticity with vortex diameter. *Positive sign* corresponds to anti-clockwise rotation and *negative sign* to clockwise rotation. The *bars* demonstrate the proportion of vortices of each sign for the semi-rigid (*blue*) and highly flexible (*red*) canopies. The *lines* plot the mean vorticity for each vortex size class, for the semi-rigid (*solid*) and highly flexible (*dotted*) canopies

Finally, we hypothesise that the medium-scale vortices relate to additional plant-flapping related turbulence within the highly flexible case. In order to investigate this further we study the relation between vortex size and vorticity for both the highly flexible and semi-rigid canopies. For vortices relating to mixing layer instabilities we expect a dominance of negative (clockwise) vorticity whereas for plant-flapping generated vortex shedding we suggest that the mean vorticity should be zero given that vortices of positive and negative vorticity are alternately shed (Fig. 11a). For each vortex scale we analyse the vorticity in the regions defined as vortices according to the Q criterion using two measures: the proportion of vortices with mean positive and negative vorticity and the mean vorticity value. The results (Fig. 14) show that the vorticity is very similar between the semi-rigid and highly flexible cases for vortices smaller than 0.07 m (small and medium scale vortices). In this region, there is a slight dominance of negative vortices (approximately 60 %) with a mean vorticity of between −1.5 and −2 s^−1^. Between 0.07 m and 0.11 m the trend is also similar, but with a greater dominance of negative vortices and correspondingly a lower mean vorticity of approximately −2.5 s^−1^. We suggest therefore that this may correspond to the most dominant mixing layer scale.

For vortices greater than 0.11 m there is a marked difference in vorticity with an increase in the dominance of negative vorticity for the semi-rigid case and the opposite for the highly flexible case. For the largest scales in the semi-rigid case the flow only consists of negative mixing layer vortices. Here the mean vorticity is approximately −5 s^−1^ though this decreases substantially at the very largest scale, suggesting a weakening of vorticity. For the highly flexible case, although the proportion of positive vortices peaks at 90 %, the mean vorticity peaks at approximately zero suggesting that the negative vortices are on average nine times stronger at this scale. This general pattern is demonstrated across the vortex diameter scale range suggesting that the mixing layer vortices are the strongest vortices within the flow and that counter-rotating vortices which we suggest relate to plant–flapping, are characterised by weaker vorticity.

## Discussion

The results presented here for both the semi-rigid and highly flexible canopies display typical canopy layer flow characteristics. This demonstrates that shear instability characteristics appear to generalise over a range of plant flexibilities [7, 85]. The normalised velocity profiles demonstrate that both canopy flows contain mixing layers associated with inflection points in the velocity profiles just above the canopy. Whilst the velocity profiles both agree with the classical mixing layer profile (particularly the highly flexible case), the Reynolds stress profiles both peak above the value observed for a classical mixing layer. This is in agreement with Sukhodolov and Sukhodolova [72] who found that for a natural vegetation canopy, the Reynolds stress profile was best described by their theoretical profile multiplied by a factor of two. The agreement with this profile observed for the highly flexible canopy (Fig. 5) suggests that the highly flexible canopy is representative of the processes occurring in the natural vegetation canopy studied by Sukhodolov and Sukhodolova [72]. For the semi-rigid case, the Reynolds stress profile exhibits an even larger peak, This is in common with the findings of Ghisalberti and Nepf [32] who observed that the magnitude of the Reynolds stress peak increased with stem rigidity, though they observed a lower magnitude peak most likely due to the lower canopy density (a = 5.2 m^−1^).

The wavelet analysis highlights the presence of mixing layer periodicities in both flows, but also suggests the presence of smaller scale, higher frequency periodicities within the highly flexible canopy flow. These periodicities do not coincide with either the wake-scale or mixing layer scale and therefore most likely relate to other turbulent production mechanisms. This observation agrees with Nikora’s [57] model for canopy flows which identifies six distinct turbulence regimes, including boundary layers, mixing layers and wakes across different scales. Of the regimes proposed, some are too large-scale (e.g. depth-scaled boundary layer, vegetated mixing layer) and others too small-scale (leaf-scale boundary layers, stem wakes) to relate to the periodicity observed in the highly flexible canopy. Therefore, we hypothesise that the observed periodicity corresponds to plant flapping induced turbulence. This mechanism cannot be simply described as one of the canonical flow types (e.g. boundary layer, mixing layer, wakes) but is most likely to be caused by a combination of, and interaction between, mixing layer instabilities and wake vortex shedding, similar to a flapping flag [86–88]. It should be noted however that a flapping flag is not the perfect analogue for vegetation stem flapping, due to it being fixed perpendicular to the flow at the bed. This mechanism of turbulence production is of great interest as it is likely to be closely related to plant form and biomechanics and will therefore vary across different plant types. Notably, this turbulence mechanism is not included within the generalised canopy layer model, where vegetation response is treated as an elastic bending response governed by the plant’s natural frequency [68, 89]. Further research is therefore required to characterise this turbulent process, assess its overall significance and contribution and to include it within the aquatic canopy flow model.

The absence of this turbulence scale (resulting from plant flapping) in the semi-rigid canopy allows a comparison of its effect in comparison to that of the mixing layer which is present in both cases. The presence of this scale does not dampen the mixing layer signal within the flow, as shown by both the normalised flow profiles and the quadrant analysis. However, there are some unexplained features which may be a result of this additional turbulence scale. The secondary peak in the Reynolds stress profile has previously been observed in canopies exhibiting coherent plant motion [8] and requires further explanation. Similarly, the highly flexible canopy exhibits a greater number of large magnitude ejection events throughout the flow depth. However, there is no corresponding increase in sweep events and therefore it is unclear as to the origin of these events. Finally, the highly flexible canopy exhibited much larger Reynolds stresses over the canopy. These phenomena require further investigation over a wider range of canopy conditions to determine the physical processes responsible for these observations and assess their persistence across a range of canopy densities, stem lengths and rigidities.

The additional turbulence production within highly flexible canopies has a clear impact on vortex characteristics. However, the impact is not straightforward. Whilst large-scale mixing layer vortices dominate the semi-rigid canopy flow, for the highly flexible canopy flow there exist large-scale vortices with positive (clockwise) vorticity. This suggests that the vortex production by plant-flapping is not restricted to the mid-scale range but also occurs at scales similar to the mixing layer vortices. It is possible that this explains the presence of two very similar low frequency scales within the wavelet plot (Fig. 8b) which split and merge through time. Neither the additional vortex occurrence at wake scales within the semi-rigid canopy, nor the additional vortex generation in the mid-scale range in the highly flexible canopy observed in Fig. 12 alter the bulk vortex characteristics as demonstrated by the similarity in Fig. 14 for scales less than 0.1 m. We suggest that this may be due to the fact that both these vortex production mechanisms generate both positive and negative vortices and therefore produce a net zero vorticity. Vortices at these smaller scales are likely to comprise both decaying mixing layer turbulence and additional turbulence production. However, the net vorticity signals of these two processes are likely to be similar. Thus we suggest that it is only mixing layer turbulence processes that significantly alter the vortex characteristics. The exception to this is at the very largest scales in the highly flexible simulation where positive vortices dominate. Here the vorticity is equal to zero suggesting the dominance of stem flapping vortices. However, the proportion of vortices that are positive is approximately 90 % rather than the 50 % expected from this vortex generation mechanism.

These results suggest a more complex picture of turbulence production within highly flexible canopies, which retains canopy mixing layer structure, but also exhibits additional turbulence production mechanisms related to stem flexibility. For highly flexible aquatic macrophytes with more complex form and foliage than considered here, we suggest that the role of this plant-flapping scale turbulence may be even further increased. However, the presence of foliage has also been shown to inhibit momentum exchange [61] and we note this as an area for future research. The turbulence generated by this mechanism has been shown to generate large-scale turbulent structures and additional high magnitude turbulent quadrant (Reynolds stress) events. Therefore, we suggest the utility of canopy-layer experiments and models employing semi-rigid or rigid vegetation analogues in drawing conclusions on flow and sediment processes in natural channels with highly flexible vegetation should be carefully considered.

Future work should be directed at evaluating the observed patterns over a wide range of canopy densities and plant forms. In order to characterise the effect of vegetation with highly complex morphology, as observed in natural environments, further model development is required to increase our capability of modelling fluid–structure interaction with increasing resolution and accuracy. This may involve more strongly coupled fluid–structure interaction models, dynamic meshing and more sophisticated turbulence models. In particular, we highlight the need to investigate the fine-scale turbulence processes operating at the wake-scale and the effect these may have on larger scale turbulence dynamics through turbulent backscatter. Nevertheless, we suggest that the methodology applied here provides a useful approach for characterising flow–vegetation interactions.

## Conclusion

This paper presents results from numerical simulations of flow through two canopies: one semi-rigid and one highly flexible. Two different models were employed to capture the dynamics of each canopy based upon their characterisation as ‘bending’ and ‘tensile’ canopies respectively. These models were applied to similar flow conditions in order to evaluate their agreement with canopy flow theory. The main conclusions of this study are:

1. The fundamentals of canopy flow generalise across a wide range of vegetation rigidities. This includes the mixing layer flow profile, vortex generation and occurrence of turbulent sweep and ejection events.
2. However, highly flexible canopies exhibit evidence of additional turbulent processes at scales that are different to those expected for mixing layers and other known turbulent processes (e.g. boundary layers and wakes).
3. These processes are most likely related to plant-flapping induced turbulence. Other than through elastic-response, such plant-related turbulent processes have not been extensively studied, but may contribute a hereto unrecognised influence on flow and channel processes in aquatic environments.
